# Association of Maternal History of Neonatal Death With Subsequent Neonatal Death in India

**DOI:** 10.1001/jamanetworkopen.2020.2887

**Published:** 2020-04-16

**Authors:** Mudit Kapoor, Rockli Kim, Tanushree Sahoo, Ambuj Roy, Shamika Ravi, A. K. Shiva Kumar, Ramesh Agarwal, S. V. Subramanian

**Affiliations:** 1 Economics and Planning Unit, Indian Statistical Institute, New Delhi, India; 2 Division of Health Policy and Management, College of Health Sciences, Korea University, Seoul, South Korea; 3 Department of Public Health Sciences, Graduate School, Korea University, Seoul, South Korea; 4 Harvard Center for Population and Development Studies, Cambridge, Massachusetts; 5 Department of Pediatrics, All India Institute of Medical Sciences, New Delhi, India; 6 Department of Cardiology, All India Institute of Medical Sciences, New Delhi, India; 7 Brookings India, New Delhi, India; 8 Development Economist and Policy Adviser, New Delhi, India; 9 Department of Social and Behavioral Sciences, Harvard T.H. Chan School of Public Health, Boston, Massachusetts

## Abstract

**Importance:**

Among the United Nations’ Sustainable Development Goals is to reduce the neonatal mortality rate to 12 per 1000 live births by 2030. Identifying high-risk pregnancies can help achieve this target in low-resource countries, such as India, which accounts for one-fourth of global neonatal deaths.

**Objective:**

To analyze the association of maternal history of neonatal death with subsequent neonatal mortality.

**Design, Setting, and Participants:**

This cross-sectional study included a nationally representative sample of singleton live births from multiparous women. Data were obtained from the 2016 National Family Health Survey in India. Data were analyzed from November 2018 to January 2020.

**Exposures:**

Maternal history of neonatal death and a comprehensive set of covariates, including socioeconomic environment, maternal anthropometry, and pregnancy care.

**Main Outcomes and Measures:**

Subsequent neonatal mortality. Population-attributable risk associated with history of neonatal death was calculated, and sensitivity analyses were performed.

**Results:**

The overall study population consisted of 127 336 singleton live births from multiparous women aged 15 to 49 (mean [SD] age, 28.8 [5.2] years) years when the survey was undertaken. In our analytic sample, 11 101 (8.7%) mothers had a history of neonatal death, and 506 of 2224 total neonatal deaths (22.8%) were attributed to women with history of neonatal death. The prevalence of history of neonatal death differed by selected covariates and across states or union territories. Maternal history of neonatal death was associated with significantly higher odds of neonatal mortality (adjusted odds ratio, 2.23; 95% CI, 1.96-2.55), and this remained consistent across different subgroups. The population-attributable risk associated with maternal history of neonatal death was 11.8%. Stronger associations were found for maternal history of multiple neonatal deaths (adjusted odds ratio, 3.50; 95% CI, 2.78-4.41) and in respect to the risk of mortality in early neonatal period (ie, 0-2 completed days) (adjusted odds ratio, 2.45; 95% CI, 2.09-2.86).

**Conclusions and Relevance:**

These findings suggest that maternal history of neonatal death is a potentially useful risk factor to identify women and neonates who may need extended and enhanced pregnancy care.

## Key Points

**Question:**

Is maternal history of neonatal death associated with subsequent neonatal mortality?

**Findings:**

In this cross-sectional study using nationally representative data of 127 336 singleton live births in India, maternal history of neonatal death was associated with more than 2-fold increased odds of subsequent neonatal mortality.

**Meaning:**

This finding suggests that maternal history of neonatal death should be part of routine antenatal assessment to identify mothers and neonates who may be at increased risk and need extended care.

## Teaser

This cross-sectional study examines the association of maternal history of neonatal death with subsequent neonatal death among multiparous women in India.

## Introduction

Reducing the neonatal mortality rate (NMR) to 12 per 1000 live births by 2030 is one of the Sustainable Development Goals of the United Nations. India accounts for more than a one-fourth of 2.6 million neonatal deaths worldwide. Global burden of disease data identified neonatal disorders as the biggest cause of premature deaths in India in 2017.^1^ Within India, there is a wide interstate variation in NMR.^2^ One immediate step toward achieving the United Nations’ Sustainable Development Goals target in a low-resource setting, such as India, is to identify risk factors associated with high NMR and conduct an early risk assessment to develop clinical and preventive programs targeted for women at the highest risk. The need for such a targeted approach has been well established since 1970s in a report by the March of Dimes.^3,4^ Subsequently, several governing bodies and the World Health Organization have adopted this approach as part of their guidelines for improving pregnancy outcomes,^5,6,7,8,9^ but many of the components either are not easily identifiable or need specific resources for detection.

Consistent evidence exists on the roles of socioeconomic conditions, fertility behaviors, low birth weight, preterm delivery, and history of adverse obstetric outcomes as important risk factors of neonatal mortality and morbidity among surviving children.^7,9,10,11,12,13,14,15^ However, in our review of experimental and observational studies that have examined the associations of obstetric history with subsequent neonatal, perinatal, or infant deaths, we found a lack of systematic evidence on the potential importance of maternal history of neonatal death. Of 29 studies that mentioned maternal history of poor pregnancy outcome as a risk factor for subsequent neonatal mortality, only 7 studies (2 studies from high- and middle-income countries,^16,17^ 2 studies from South East Asia,^18,19^ and 3 studies from Africa^20,21,22^) have specifically found that history of neonatal death was a significant risk factor. However, these studies were based on small populations (ie, not nationally representative) and controlled for a limited number of confounding variables.

Perhaps owing to such lack of systematic evidence, maternal history of neonatal death remains absent from World Health Organization guidelines concerning high-risk pregnancy.^5,6,7,8,9^ While women with a history of neonatal death are identified as a high-risk group in the *International Classification of Diseases, 11th Revision*,^23^ it is not incorporated in governmental health programs in low- and middle-income countries. In the Indian context, for instance, maternal history of neonatal death is not featured in the Pradhan Mantri Surakshit Matritva Abhiyan,^8^ a flagship program launched in 2016 to provide comprehensive and high-quality antenatal care, free of cost, to all pregnant women on the ninth day of every month. One of the factors outlined in the Pradhan Mantri Surakshit Matritva Abhiyan to identify high-risk pregnancy is history of adverse obstetric outcomes, including history of still birth, abortion, congenital malformation, obstructed labor, and premature birth.^8^ However, the documentation in Pradhan Mantri Surakshit Matritva Abhiyan exclusively focuses on previous cesarean birth, followed by a recommendation to ensure birth in a comprehensive emergency obstetric care setting, and there is no explicit mention of history of neonatal death. A key advantage of using history of neonatal death as a screening tool to identify women at higher risk of neonatal mortality is its practicality and applicability for individual clinical care as well as targeted public health policies and interventions.

In this study, we performed comprehensive analyses using the latest nationally representative data from India to test our hypothesis that maternal history of neonatal death is an important risk factor for subsequent neonatal mortality, conditional on diverse factors that range from socioeconomic environment to maternal anthropometry and pregnancy care.

## Methods

This study used an anonymous public use data set without identifiable information about individuals in the study. As such, it was considered exempt from full review and informed consent by the Harvard T.H. Chan School of Public Health Institutional Review Board. This study follows the Strengthening the Reporting of Observational Studies in Epidemiology (STROBE) reporting guideline.

### Data Source and Study Population

We used data from the fourth round of the National Family Health Survey (NFHS-4), a large-scale nationally representative survey of rural and urban Indian households. The NFHS-4 was conducted in 2015 to 2016 with a total of 699 686 women aged 15 to 49 years from 29 Indian states and 7 union territories across all 640 districts in India. Census enumeration blocks in urban areas and villages in rural areas served as the primary sampling units for NFHS-4. For each census enumeration block or village, the sampling frame contained information about locations, such as state, district, type of residence (rural or urban), estimated number of residential households and population, and percentage of population belonging to scheduled caste or scheduled tribe. Details regarding the survey objective, sample design, questionnaires, biomarkers measured, and other details have been published elsewhere.^24^ Using computer-assisted programming interviewing, interviewers obtained complete birth history from every woman surveyed, including date of birth, birth order, sex of the child, singleton or multiple birth, whether the child was alive or dead, age at death, and current age of the living child. In addition, a biomarker questionnaire was also administered to obtain measures of anthropometry, hemoglobin, blood pressure, and blood glucose. For our analysis, we limited the final sample to the most recent singleton live births in the 5 years before the survey from multiparous women.

### History of Neonatal Death

Our primary risk factor was maternal history of neonatal death, defined by examining the complete birth history of the mother, excluding her most recent live birth. A binary variable was constructed with a value of 1 if the mother had a prior live birth that resulted in neonatal death, and 0 if not. We also defined 2 alternative risk factors: postneonatal mortality, defined as history of infant death in the period between 28 days and less than 1 year, and child mortality, defined as history of death in the period between ages 1 and 4 years.

### Outcomes

Our primary outcome was neonatal mortality, defined as death between 0 to 27 completed days, in the most recent live birth in the 5 years before the survey. Secondary outcomes were mortality in the most recent live births in different neonatal periods: 0 to 2 completed days, 3 to 6 completed days, and 7 to 27 completed days.

### Covariates

We adjusted for several factors, ranging from socioeconomic environment to maternal anthropometry and pregnancy care, that are known to be associated with neonatal mortality (eTable 1 in the Supplement). To minimize loss of information and potential selection bias, a separate category was created for missing data or for do not know responses for all variables.

The socioeconomic environmental covariates included residence (urban or rural), maternal education (ie, no schooling, <5 years, 5-7 years, 8-9 years, 10-11 years, or ≥12 years), wealth status (in quintiles), status of insurance coverage, religion (ie, Hindu, Muslim, Christian, Sikh, Buddhist or Dalit Buddhist, or other), caste (ie, scheduled caste, scheduled tribe, other backward class, or other), sanitary facility (ie, flush toilet, pit latrine, open defecation, or other), and water supply for drinking and domestic purposes (ie, piped water; public tap or standpipe; tube well or borewell; protected or unprotected well; protected or unprotected spring; river, dam, lake, pond, stream, or canal; rain water; tanker truck; cart with small tank; bottled water; community reverse osmosis water purifying plant; other; or not a de jure resident). Maternal factors included age at birth (ie, <18 years, 18-34 years, or ≥35 years), height (<145 cm, 145-149.9 cm, 150-154.9 cm, 155-159.9 cm, or ≥160 cm), body mass index (calculated as weight in kilograms divided by height in meters squared) at the time of the interview (ie, <16.5, 16.5-18.49, 18.5-24.99, and ≥25), tobacco use, and alcohol consumption. Pregnancy-related factors included anemia at the time of the interview (classified using hemoglobin levels and defined as severe: <7 g/dL; moderate: 7-9.9 g/dL; mild: 10-10.9 g/dL; or not anemic: ≥11 g/dL [to convert to grams per liter, multiply by 10]); hypertension (based on the mean of second and third readings, women were considered hypertensive if their systolic blood pressure was ≥140 mm Hg or diastolic blood pressure was ≥90 mm Hg); blood glucose level (ie, normal: <140 mg/dL, and high: ≥140 mg/dL [to convert to millimoles per liter, multiply by 0.0555]); pregnancy duration (ie, <9 months or ≥9 months); birth order (ie, 2-3, 4-6, ≥7); previous pregnancy loss (ie, miscarriage, abortion, or stillbirth); mode of delivery (ie, cesarean or vaginal); birth weight of baby stratified by type of report (ie, not weighed, <2500 g based on written card, <2500 g based on mother’s recall, ≥2500 g based on written card, or ≥2500 g based on mother’s recall); baby’s size at birth (ie, within reference range or larger, small, or very small); and interpregnancy interval (ie, <18 months, 18-59 months, or ≥60 months from the previous birth).

Antenatal care factors included frequency of antenatal care visits (ie, none, 1-4, 5-7, 8-9, or ≥10); full tetanus protection; presence of skilled birth assistant at time of delivery; place of delivery (ie, at home or other); use of mosquito nets; history of convulsions from fever; presence of edema; swelling of the legs, body, or face; provision of supplementary nutrition (from anganwadi center or rural child care center); difficulty in getting medical help owing to distance from the facility, monetary help for the treatment, facility for safe transport, presence of companion during transport, absence of women health care practitioners, or nonavailability of drugs; regular measurements or tests of weight, blood pressure, urine, and blood during antenatal visit; and appraisal about pregnancy complications (eg, vaginal bleeding, convulsions, prolonged labor, severe abdominal pain, high blood pressure).

### Statistical Analysis

First, we assessed the prevalence of mothers with history of neonatal death by covariates and across 36 states and union territories. To assess the association of mothers with history of neonatal death and subsequent neonatal mortality, we performed a series of unadjusted and adjusted logistic regression models. The consistency in this association was evaluated in several stratified analyses, with subgroups defined by (1) household wealth, (2) states with estimated NMR of more than 30 deaths per 1000 live births (ie, Assam, Bihar, Chhattisgarh, Madhya Pradesh, and Uttar Pradesh), (3) states with estimated NMR of 20 deaths or fewer per 1000 live births (ie, Karnataka, Kerala, Maharashtra, Tamil Nadu, and Telangana), (4) sex of the child, (5) frequency of antenatal care visits, (6) whether the mother had full tetanus protection, (7) maternal intake of iron-folic tablets or syrup, (8) place of delivery, (9) birth interval, (10) birth weight, and (11) mother’s age at birth, all of which are considered highly relevant for public policies aimed at reducing NMR in low- to middle-income countries. We also computed the risk of mortality associated with maternal history of neonatal death using the entire sample of 190 898 most recent live births from the NFHS-4 data.

Several sensitivity analyses were performed. First, we divided our key risk factor, mothers with history of neonatal death, into whether the mother’s history included a single neonatal death or 2 or more neonatal deaths. Second, we reassessed the logistic regression models using alternative definitions of history of death in postneonatal periods (ages 28 days to 1 year) and childhood periods (ages 1 to 4 years). Third, we assessed the association of maternal history of neonatal death with secondary outcomes of neonatal mortality occurring between 0 to 2, 3 to 6, and 7 to 27 completed days.

For all analyses, we included state fixed effects to account for unobserved regional differences, which might be correlated with other associated variables. Our estimation strategy accounted for the sampling design in which sample weights were used in the estimation of the coefficients. Errors were clustered at the primary sampling units to account for potential correlations between observations within the same primary sampling unit, and they were computed using the linearization-based variance estimators. Strata with a single sampling unit were treated with certainty. All analyses were performed in Stata statistical software version 14.2 (StataCorp). *P* values were 2-tailed, and statistical significance was set at .05. Data analysis began November 2018, and final data analysis was completed in January 2020.

## Results

**Figure 1.**
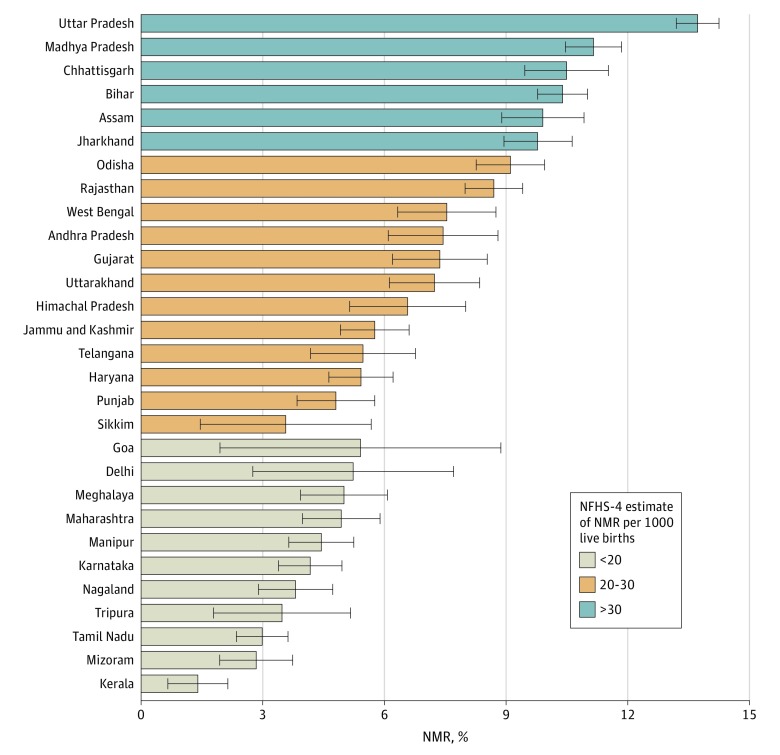
Interstate Variations in Proportion of Mothers With History of Neonatal Death in India Error bars indicate 95% CI; NFHS-4, National Family Health Survey; NMR, neonatal mortality rate.

Of 190 898 live births recorded, we excluded 61 807 observations on live births from nulliparous women and 1755 nonsingleton live births. A total of 127 336 live births from multiparous women aged 15 to 49 (mean [SD] age, 28.8 [5.2] years) years were included in our primary analysis. Among these, 11 101 mothers (8.7%) had histories of neonatal death, and this group contributed 506 of the total of 2224 neonatal deaths recorded (22.8%). The unadjusted NMR among mothers with history of neonatal death was statistically significantly higher compared with those without history of neonatal death (47.85 per 1000 live births vs 14.13 per 1000 live births; *P* < .001) (Table). Mothers with history of neonatal death were more likely than mothers without history of neonatal death to be poorer (4453 women [40.1%] vs 30 398 women [26.2%]; *P* < .001), have no schooling (5436 women [49.0%] vs 38 927 women [33.5%]; *P* < .001), have received no antenatal care (2660 women [24.0%] vs 22 562 women [19.4%]; *P* < .001), have a neonate not weighed at birth (3266 women [29.4%] vs 23 421 women [20.2%]; *P* < .001), have a preterm delivery (867 women [7.8%] vs 7432 women [6.4%]; *P* < .001), have a birth interval less than 18 months (2117 women [19.1%] vs 10 143 women [8.7%]; *P* < .001), and have a neonate who had very small birth size (489 women [4.4%] vs 3082 women [2.7%]; *P* < .001) (Table). We also found significant interstate variations in the proportion of live births among women with history of neonatal death, ranging from 13.7% (95% CI, 13.2%-14.2%) in Uttar Pradesh to 1.4% (95% CI, 0.6%-2.1%) in Kerala (Figure 1).

**Table.** Characteristics of the Multiparous Women Stratified by History of Neonatal Death

Characteristic	History of neonatal death, No. (%)		
	Yes (n = 11 101)	No (n = 116 235)	*P* value
Neonatal mortality per 1000 live births^a^			
Unadjusted	47.9	14.1	<.001
Adjusted	31.9	15.1	<.001
Unadjusted early	41.7	11.6	<.001
Adjusted early	27.9	12.4	<.001
Wealth, quintile			
Poorest	4453 (40.1)	30 398 (26.2)	<.001
Poorer	2778 (25.0)	25 602 (22.0)	<.001
Middle	1908 (17.2)	23 154 (19.9)	<.001
Richer	1277 (11.5)	20 885 (18.0)	<.001
Richest	684 (6.2)	16 196 (13.9)	<.001
Mother’s schooling, y			
0	5436 (49.0)	38 927 (33.5)	<.001
<5	902 (8.1)	7659 (6.6)	<.001
5-7	1808 (16.3)	19 929 (17.2)	.10
8-9	1419 (12.8)	18 486 (15.9)	<.001
10-11	710 (6.4)	13 412 (11.5)	<.001
≥12	826 (7.4)	17 822 (15.3)	<.001
Antenatal care visits, No.			
0	2660 (24.0)	22 562 (19.4)	<.001
1-4	5393 (48.6)	49 972 (43.0)	<.001
5-7	1725 (15.5)	22 617 (19.5)	<.001
8-9	727 (6.6)	11 222 (9.7)	<.001
≥10	501 (4.5)	8907 (7.7)	<.001
Birth weight^b^			
Not weighed	3266 (29.4)	23 421 (20.2)	<.001
Low			
Mother’s recall	824 (7.4)	6660 (5.7)	<.001
Written card	724 (6.5)	7643 (6.6)	.88
Within reference range or higher			
Mother’s recall	2901 (26.1)	31 921 (27.5)	.02
Written card	2914 (26.3)	43 357 (37.3)	<.001
Duration of pregnancy, mo			
<9	867 (7.8)	7432 (6.4)	<.001
≥9	10 208 (92.0)	108 729 (93.5)	<.001
Birth interval, mo			
<18	2117 (19.1)	10 143 (8.7)	<.001
18-59	7802 (70.3)	87 728 (75.5)	<.001
≥59	1182 (10.7)	18 364 (15.8)	<.001
Mother’s age at birth, y			
<18	66 (0.6)	595 (0.5)	.42
18-34	9745 (87.8)	108 808 (93.6)	<.001
≥35	1290 (11.6)	6832 (5.9)	<.001
Birth size			
Within reference range or larger	9161 (82.5)	101 451 (87.3)	<.001
Small	1166 (10.5)	9774 (8.4)	<.001
Very small	489 (4.4)	3082 (2.7)	<.001

^a^ Estimated by computing the mean predicted probabilities after running a logistic regression with neonatal and early neonatal mortality as the dependent variables and history of neonatal death as the key explanatory variable. The adjusted model is further adjusted for factors related to socioeconomic environment, maternal anthropometry, and pregnancy care.

^b^ Reference range birth weight was defined as 2500 grams or more.

**Figure 2.**
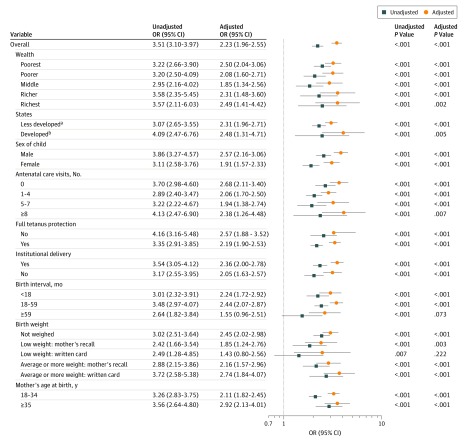
Adjusted Odds Ratios (ORs) for Neonatal Mortality for Overall Sample and Subgroup Stratified by Maternal History of Neonatal Death

In our main analysis, we found a significant association of maternal history of neonatal death with neonatal mortality in univariate and multivariate logistic regression models. History of neonatal death was associated with 3.51 (95% CI, 3.10-3.97) higher odds of neonatal mortality before adjusting for other covariates and 2.23 (95% CI, 1.96 to 2.55) higher odds of neonatal mortality after adjusting for other covariates (Figure 2; eTable 2 in the Supplement). A consistent association was found across all stratified analyses, except for subgroups of mothers with birth intervals 59 months or longer (adjusted odds ratio [aOR], 1.55 [95% CI, 0.96-2.51]; *P* = .07) and low birth weight based on written card (aOR, 1.43 [95% CI, 0.80-2.50]; *P* = .22). The association of maternal history of neonatal death with neonatal mortality was statistically significant in states with higher NMRs (aOR, 2.31 [95% CI, 1.96-2.71]; *P* < .001) and states with lower NMRs (aOR, 2.48 [95% CI, 1.31-4.71]; *P* < .001). The population-attributable risk for history of neonatal death was estimated to be 11.8% (eTable 3 in the Supplement).

**Figure 3.**
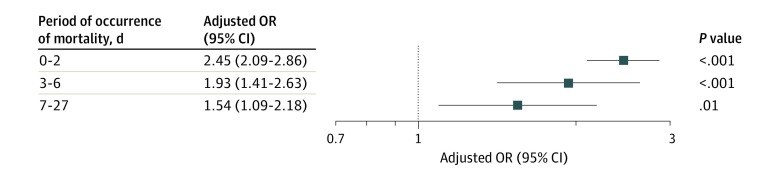
Unadjusted and Adjusted Odds Ratio (OR) for Period of Occurrence of Mortality Stratified by Maternal History of Neonatal Death

Our main findings remained robust across several sensitivity analyses. First, we found that mothers who had a history of 2 or more neonatal deaths had a significantly higher aOR of neonatal mortality (aOR, 3.50 [95% CI, 2.78-4.41]) compared with mothers with history of 1 neonatal death (aOR, 2.01 [95% CI, 1.74-2.32]) (eFigure 1 in the Supplement). Second, we noted that history of death in the postneonatal period (ie, ages 28 days to 1 year) and childhood period (ie, ages 1-4 years) were not significantly associated with subsequent neonatal mortality (eFigure 2 in the Supplement). Lastly, the association of maternal history of neonatal death with subsequent mortality was stronger for earlier periods of life, with aORs of 2.45 (95% CI, 2.09-2.86) for deaths within 0 to 2 completed days, 1.93 (95% CI, 1.41-2.63) for deaths within 3 to 6 completed days, and 1.54 (95% CI, 1.09-2.18) for deaths within 7 to 27 completed days (Figure 3).

## Discussion

This cross-sectional study had 4 salient findings from our analysis of large-scale, nationally representative data in India. First, we detected differential prevalence of mothers with history of neonatal death by selected covariates and across states and union territories, indicating the modifiability of this exposure. Second, we found robust evidence of an association of maternal history of neonatal death with increased risk of subsequent neonatal mortality during the study, after adjusting for a comprehensive set of covariates. Moreover, this association remained statistically significant across different subgroups defined by other known socioeconomic and pregnancy-related risk factors of neonatal mortality. Third, we found that maternal history of multiple neonatal deaths was associated with even higher odds of subsequent neonatal mortality. Lastly, the association of mothers with history of neonatal death with the risk of neonatal mortality observed during the study was strongest for the early neonatal period (ie, 0-2 completed days), suggesting the need for intense postnatal follow-up and care immediately after birth for neonates born to mothers with a history of neonatal death.

One potential explanation for the observed association is that many of the factors that have been linked to neonatal mortality in pregnancy (ie, socioeconomic conditions, access to proper care during antenatal and intrapartum periods, and pregnancy-related complications) are likely to persist and recur in subsequent pregnancies. Adjusting for a comprehensive set of covariates resulted in a substantial attenuation in the association of maternal history of neonatal death with subsequent neonatal mortality observed during the study, but the association retained statistical significance and the magnitude of the association remained large after covariate adjustment. We speculate that this higher predisposition to neonatal mortality in the presence of history of neonatal death may reflect residual confounding or some inherent maternal or neonatal factors associated with less common and difficult to diagnose genetic, immunological, or other complex disorders. For instance, owing to data-related limitations, we were unable to control for the quality of care provided for different pregnancy and neonatal complications, which may partly explain the higher risk of neonatal mortality.

Several studies have identified various risk factors for neonatal mortality, including low socioeconomic status, lower education, inadequate antenatal care, low birth weight, premature birth, and obstetric or intrapartum complications.^7,9,10,11,12,13,14,15^ However, only a few studies have analyzed the association of mothers with history of neonatal death with subsequent neonatal mortality.^10,16,17,18,19,20,21,22^ In a regional study restricted to 2 rural districts of Uttar Pradesh in North India, Williams et al^18^ found history of neonatal death to be an important risk factor for subsequent neonatal death. Similar to our sensitivity analysis, Williams et al also noted that the risk of neonatal death increased for maternal history of multiple neonatal deaths. Another retrospective study from Matlab, a subdistrict in Bangladesh,^19^ reported neonates with previous history of sibling death during the neonatal period to be at approximately 2-fold higher risk of dying compared with their counterparts without such history. Although these studies used large data sets, they were regional studies, conducted retrospectively, and adjusted for a smaller number of confounders, and they found weaker associations compared with our study.

Our finding on the higher risk associated with maternal history of neonatal death especially for early neonatal mortality (ie, deaths within 0-2 completed days) is consistent with literature hypothesizing different causes of deaths operating at different neonatal periods. Pathways and associations concerning different risk factors are often complicated and poorly understood. Typically, pregnancy- and labor-related factors are expected to exert a greater importance for early neonatal deaths than for late neonatal or postneonatal deaths. However, in respect to subsequent neonatal mortality, our analysis found differential association for history of neonatal death vs past pregnancies terminated in abortions, miscarriage, and stillbirths. One possibility could be underreporting of past pregnancies that resulted in abortions, miscarriages, and stillbirths owing to cultural beliefs.^25,26^

### Limitations

There are a few limitations to consider in interpreting our findings. Ideally maternal history of previous stillbirths should have been combined with history of neonatal death to examine the overall burden of history of adverse obstetric outcomes because many important risk factors of stillbirths and early neonatal deaths are similar. However, the NFHS-4 data did not specify stillbirths as a separate category in the questionnaire. Although we accounted for many covariates to minimize the confounding bias, we cannot completely rule out the possibility of residual confounding or bias due to unknown confounders. Given the cross-sectional nature of this study, we do not claim causality in the observed associations. Further research with stronger study designs is needed to assess the causality and underlying mechanisms between maternal history of neonatal death and subsequent neonatal mortality.

## Conclusions

This cross-sectional study found associations of mothers with history of neonatal death with early neonatal mortality and overall neonatal mortality during this study in India. These findings have important implications for clinicians as well as policy makers. For clinicians, maternal history of neonatal death may serve as an important marker for identifying and managing high-risk pregnancies as well as neonates born in such condition. The precise cause of recurrence for such neonatal deaths should be delineated in future in-depth research. Nevertheless, our findings, when interpreted in light of prior evidence, also suggest policy makers should incorporate the presence of history of neonatal death as one of the criteria for identifying high-risk pregnancies.

## Notes

Supplement. **eFigure 1.** Adjusted Odds Ratios for Neonatal Mortality Stratified by Maternal History of 1 or More Than 1 Neonatal Death **eFigure 2.** Adjusted Odds Ratio for Neonatal Mortality Stratified by Maternal History of Death or no History of Death in Different Periods **eTable 1.** Characteristics of the Live Births Stratified by Maternal History of Neonatal Death **eTable 2.** Adjusted Odds Ratios for Neonatal and Early Neonatal Mortality **eTable 3.** Maternal History of Neonatal Death and the Attributable and Population–Attributable Risk of Mortality
