# Supplementary material for: Association of Maternal History of Neonatal Death With Subsequent Neonatal Death in India
Source: JAMA Netw Open. 2020 Apr 16;3(4):e202887. doi: 10.1001/jamanetworkopen.2020.2887 (PMC7163408; doi:10.1001/jamanetworkopen.2020.2887)
Supplement: Supplement. — eFigure 1. Adjusted Odds Ratios for Neonatal Mortality Stratified by Maternal History of 1 or More Than 1 Neonatal Death eFigure 2. Adjusted Odds Ratio for Neonatal Mortality Stratified by Maternal History of Death or no History of Death in Different Periods eTable 1. Characteristics of the Live Births Stratified by Maternal History of Neonatal Death eTable 2. Adjusted Odds Ratios for Neonatal and Early Neonatal Mortality eTable 3. Maternal History of Neonatal Death and the Attributable and Population–Attributable Risk of Mortality [file jamanetwopen-3-e202887-s001.pdf]

Supplementary Online Content

Kapoor M, Kim R, Sahoo T, et al. Association of maternal history of neonatal death with subsequent neonatal death in India. *JAMA Netw Open*. 2020;3(4):e202887. doi:10.1001/jamanetworkopen.2020.2887

eFigure 1. Adjusted Odds Ratios for Neonatal Mortality Stratified by Maternal History of 1 or More Than 1 Neonatal Death

eFigure 2. Adjusted Odds Ratio for Neonatal Mortality Stratified by Maternal History of Death or no History of Death in Different Periods

eTable 1. Characteristics of the Live Births Stratified by Maternal History of Neonatal Death

eTable 2. Adjusted Odds Ratios for Neonatal and Early Neonatal Mortality

eTable 3. Maternal History of Neonatal Death and the Attributable and Population–Attributable Risk of Mortality

This supplementary material has been provided by the authors to give readers additional information about their work.

© 2020 Kappor M et al. *JAMA Network Open.*

eFigure 1. Adjusted Odds Ratios for Neonatal Mortality Stratified by Maternal History of 1 or More Than 1 Neonatal Death

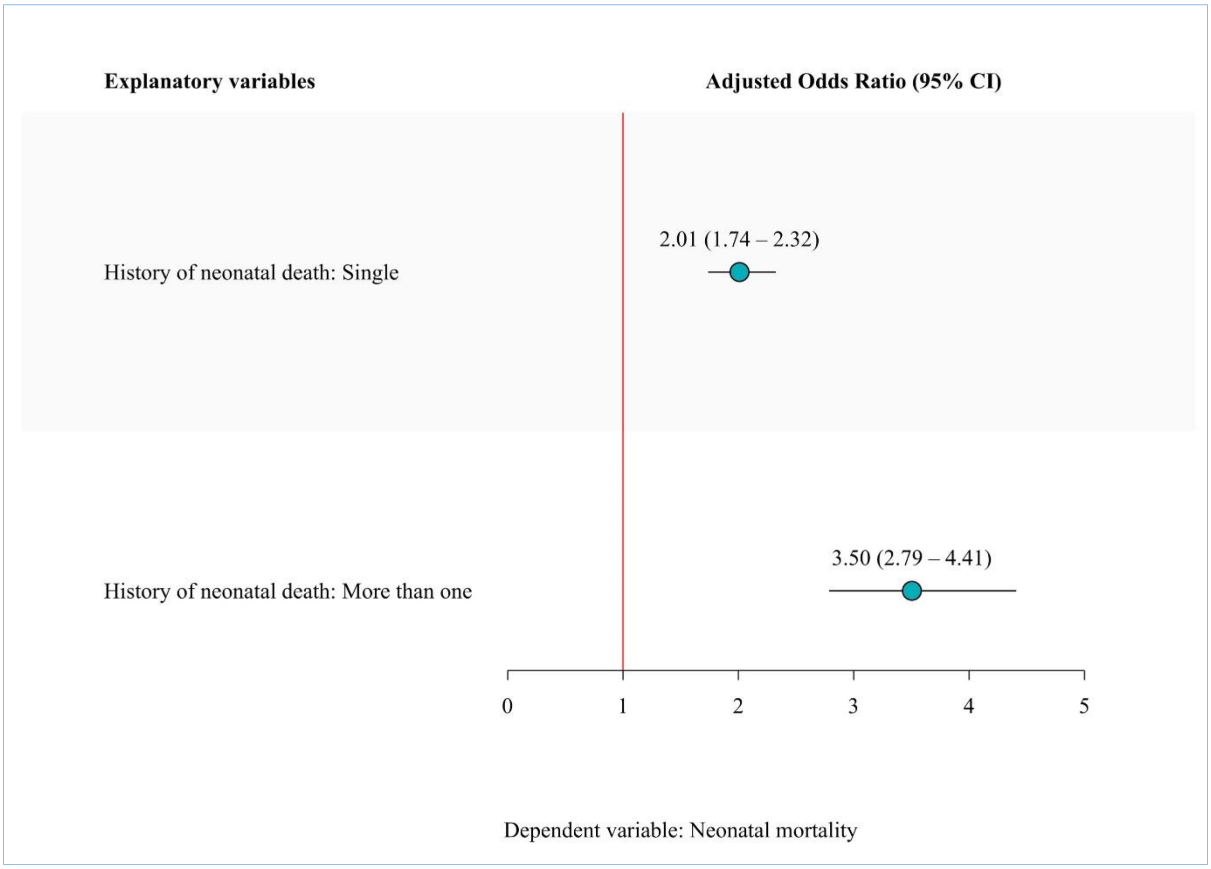

The adjusted odds ratios is adjusted for factors related to socioeconomic environment, maternal anthropometry, and pregnancy care. The exhaustive list of variables used in the analysis are presented in the supplementary table S1.

© 2020 Kappor M et al. *JAMA Network Open.*

eFigure 2. Adjusted Odds Ratio for Neonatal Mortality Stratified by Maternal History of Death or no History of Death in Different Periods

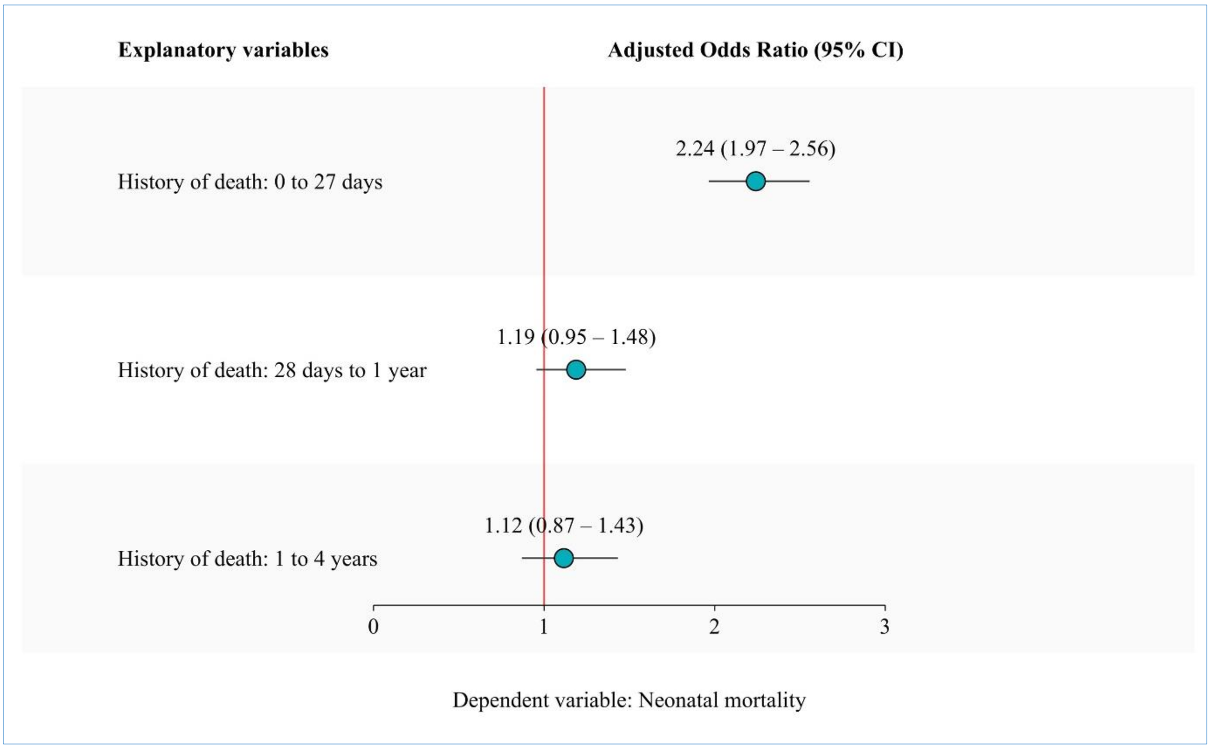

The adjusted model is adjusted for factors related to socioeconomic environment, maternal anthropometry, and pregnancy care. The exhaustive list of variables used in the analysis are presented in the supplementary table S1.

© 2020 Kappor M et al. *JAMA Network Open.*

eTable 1. Characteristics of the Live Births Stratified by Maternal History of Neonatal Death

**List of variables**	History of neonatal death<br>Yes<br>(N = 11,101)	 No<br>(N = 116,235)	p Value
Mortality*			
Unadjusted neonatal mortality (per 1000 livebirths)	47.9	14.1	< 0.001
Unadjusted early neonatal mortality (per 1000 livebirths)	41.7	11.6	< 0.001
Wealth (%)			
Poorest	40.1	26.2	< 0.001
Poorer	25.0	22.0	< 0.001
Middle	17.2	19.9	< 0.001
Richer	11.5	18.0	< 0.001
Richest	6.2	13.9	< 0.001
Mother's schooling in Years (%)			
No Schooling	49.0	33.5	< 0.001
Less than 5 Years	8.1	6.6	< 0.001
5 to 7 years	16.3	17.2	0.10
8 to 9 years	12.8	15.9	< 0.001
10 to 11 years	6.4	11.5	< 0.001
12 or more years	7.4	15.3	< 0.001
Frequency of ANC Visits (%)			
No Antenatal Visits	24.0	19.4	< 0.001
1 to 4 visits	48.6	43.0	< 0.001
5 to 7 visits	15.5	19.5	< 0.001
8 to 9 visits	6.6	9.7	< 0.001
10 or more visits	4.5	7.7	< 0.001
Don't know	0.6	0.8	0.17
Missing	0.2	0.1	0.003
Birth weight (%)			
Not weighed	29.4	20.2	< 0.001
Low weight: mother's recall	7.4	5.7	< 0.001
Low weight: written card	6.5	6.6	0.88
Average or more weight: mother's recall	26.1	27.5	0.02
Average or more weight: written card	26.3	37.3	< 0.001
Don't Know/Missing	4.3	2.8	< 0.001
Duration of Pregnancy (%)			
Less than 9 months	7.8	6.4	< 0.001
9+ months	92.0	93.5	< 0.001
Missing	0.2	0.1	0.003
Birth interval (months) (%)			
Less than 18 months	19.1	8.7	< 0.001
18 to 59 months	70.3	75.5	< 0.001
59 or more months	10.7	15.8	< 0.001
Mother's Age at Birth (%)			
Less than 18 Years	0.6	0.5	0.42
18 to 34 years	87.8	93.6	< 0.001
35 or more years	11.6	5.9	< 0.001
Birth Size (%)			
Average or Larger	82.5	87.3	< 0.001
Small	10.5	8.4	< 0.001
Very Small	4.4	2.7	< 0.001
Don't Know	2.3	1.6	< 0.001
Missing	0.2	0.1	0.003
Religion (%)			
Hindu	79.8	77.9	< 0.001
Muslim	17.3	17.3	0.98
Christian	1.3	2.1	< 0.001

© 2020 Kappor M et al. *JAMA Network Open.*

Sikh	0.6	1.1	< 0.001
Buddhist / Neo- Buddhist	0.3	0.8	< 0.001
Other	0.7	0.8	0.40
Caste (%)			
Scheduled Caste	24.7	21.5	< 0.001
Scheduled Tribe	11.0	10.6	0.29
Other Backward Caste (OBC)	44.8	44.3	0.49
Other	18.5	22.8	< 0.001
Don't Know	1.0	0.9	0.39
Residence (%)			
Urban	17.8	28.3	< 0.001
Rural	82.3	71.7	< 0.001
Covered by Health Insurance (%)			
Yes	13.7	15.2	0.003
Toilet Facility (%)			
Flush	30.2	42.5	< 0.001
Pit Latrine	5.8	6.6	0.04
Open Defecation	59.0	45.3	< 0.001
Other	4.9	5.7	0.008
Source of Drinking Water (%)			
Piped into dwelling	7.0	12.4	< 0.001
Piped to yard/plot	7.9	11.3	< 0.001
Public tap/standpipe	10.7	12.9	< 0.001
Tube well or borehole	59.9	45.4	< 0.001
Protected well	1.4	2.7	< 0.001
Unprotected well	4.8	4.6	0.33
Protected Spring	0.2	0.3	0.007
Unprotected Spring	0.4	0.5	0.02
River/Dam/Lake/Ponds/Stream/Canal	0.6	0.8	0.06
Rainwater	0.2	0.2	0.69
Tanker Truck	1.2	1.4	0.39
Cart with Small Tank	0.1	0.1	0.70
Bottled Water	1.1	2.3	< 0.001
Community RO Plant	0.4	0.4	0.96
Other	0.1	0.1	0.90
Not a de jure resident	4.1	4.8	0.01
Smoke/Tobacco (%)			
Yes	10.4	6.7	< 0.001
Alcohol (%)			
Yes	1.6	1.2	0.0023
Skilled Birth Assistance (%)			
Yes	71.8	78.9	< 0.001
Missing	0.2	0.1	0.003
Delivery at Home or Other (%)			
Yes	30.5	23.4	< 0.001
Full tetanus protection (%)			
Yes	87.9	88.5	0.15
Took Iron Tablets and Syrup for 100 days or more (%)			
No iron tablets/syrup	29.5	24.4	< 0.001
Less than 100	47.1	46.1	0.13
100+	21.9	27.9	< 0.001
Don't know	1.3	1.5	0.17
Missing	0.2	0.1	0.003
Took drugs for intestinal parasites during pregnancy (%)			
Yes	12.9	17.3	< 0.001
Don't know	1.0	0.9	0.15
Missing	0.2	0.1	0.003
During Pregnancy: Mosquito Net Usage (%)			
Never	45.7	46.8	0.09
Sometimes	19.5	20.8	0.01

© 2020 Kappor M et al. *JAMA Network Open.*

Regularly	34.9	32.4	< 0.001
During Pregnancy: Convulsions not from Fever (%)			
Yes	20.9	16.5	< 0.001
Don't Know	0.1	0.1	0.16
During Pregnancy: Swelling of legs, body or face (%)			
Yes	32.2	29.9	< 0.001
Don't Know	0.0	0.1	0.34
During Pregnancy: Received Supplementary Nutrition from Anganwadi Centre (%)			
Yes	56.1	55.8	0.72
Getting Medical Help for self: Getting Permission to go (%)			
No Problem	57.2	55.9	0.06
Big Problem	21.1	20.4	0.22
Not a big Problem	21.7	23.6	< 0.001
Getting Medical Help for self: Getting money needed for treatment (%)			
No Problem	37.1	40.8	< 0.001
Big Problem	32.9	28.2	< 0.001
Not a big Problem	30.0	31.0	0.11
Getting Medical Help for self: Distance to health facility (%)			
No Problem	26.5	31.7	< 0.001
Big Problem	39.3	33.7	< 0.001
Not a big Problem	34.2	34.6	0.52
Getting Medical Help for self: Having to take transport (%)			
No Problem	28.7	34.8	< 0.001
Big Problem	37.7	31.3	< 0.001
Not a big Problem	33.6	34.0	0.60
Getting Medical Help for self: Not wanting to go alone (%)			
No Problem	39.8	43.4	< 0.001
Big Problem	26.6	22.9	< 0.001
Not a big Problem	33.6	33.7	0.85
Getting Medical Help for self: Concern no female health provider (%)			
No Problem	26.1	29.5	< 0.001
Big Problem	45.2	41.1	< 0.001
Not a big Problem	28.7	29.3	0.29
Getting Medical Help for self: Concern no provider (%)			
No Problem	23.4	26.3	< 0.001
Big Problem	53.5	48.9	< 0.001
Not a big Problem	23.2	24.8	0.005
Getting Medical Help for self: Concern no drugs available (%)			
No Problem	24.3	27.5	< 0.001
Big Problem	54.8	50.2	< 0.001
Not a big Problem	21.0	22.3	0.018
During Pregnancy: Weighed (%)			
Yes	63.3	71.7	< 0.001
During Pregnancy: Blood Pressure taken (%)			
Yes	61.6	70.5	< 0.001
During Pregnancy: Urine Sample taken (%)			
Yes	60.1	69.1	< 0.001
During Pregnancy: Blood Sample taken (%)			
Yes	59.0	68.4	< 0.001
Told about Pregnancy Complication: Vaginal bleeding (%)			
Yes	29.2	35.6	< 0.001
Told about Pregnancy Complication: Convulsions (%)			
Yes	28.6	34.1	< 0.001
Told about Pregnancy Complication: Prolonged Labour (%)			
Yes	32.2	39.0	< 0.001
Told about Pregnancy Complication: Severe Abdominal Pain (%)			
Yes	33.9	40.3	< 0.001

© 2020 Kappor M et al. *JAMA Network Open.*

Told about Pregnancy Complication: High Blood Pressure (%)			
Yes	33.1	40.0	< 0.001
Past pregnancy ended in abortion, miscarriage or stillbirth (%)			
Yes	18.9	16.8	< 0.001
Birth Order (%)			
2 to 3	44.2	80.2	< 0.001
4 to 6	44.9	17.8	< 0.001
7 or more	11.0	2.1	< 0.001
Mother's Height (%)			
Less than 145 cm	17.9	11.3	< 0.001
145 to 149.9 cm	30.1	26.6	< 0.001
150 to 154.9 cm	30.6	33.5	< 0.001
155 to 159.9 cm	15.6	19.5	< 0.001
160 or more cm	4.5	7.4	< 0.001
Refused	0.6	0.9	0.06
Other	0.1	0.1	0.70
Missing	0.6	0.7	0.40
BMI Cut off (kg/cm^2) (%)			
Less than 16.5	6.7	5.8	0.006
16.5 to 18.49	20.8	17.9	< 0.001
18.5 to 24.99	58.6	58.6	0.99
25 or more	12.6	15.9	< 0.001
Flagged	1.5	1.8	0.08
Hypertension[i] (%)			
Yes	6.4	5.1	< 0.001
Missing/Refused/Technical Problem	3.9	4.6	0.01
Anaemia[ii] (%)			
Severe	1.5	1.0	0.001
Moderate	15.5	13.8	< 0.001
Mild	39.9	41.3	0.03
None	41.4	41.7	0.56
Missing	1.7	2.2	0.0277
Blood sugar (%)			
140 mg/dl or less	94.4	94.2	0.46
More than 140 mg/dl	3.8	3.6	0.26
Refused/Other/Not tested	1.2	1.6	0.06
Missing	0.6	0.7	0.29
Delivery by Caesarean Section (%)			
Yes	13.1	15.0	< 0.001
Sex of Child (%)			
Male	52.5	55.2	< 0.001
Female	47.5	44.8	< 0.001

[i] Hypertensive: Average of the 2nd and 3rd reading – Systolic more than 140mm of Hg, and/or Diastolic more than 90mm of Hg.

[ii] Anaemia adjusted for altitude and for smoking status. Severe: Hemoglobin less than 7 grams per deciliter (g/dl). Moderate: 7 to 9.9 g/dl. Mild: 10 to 10.9 g/dl. None: 11 or more g/dl.

© 2020 Kappor M et al. *JAMA Network Open.*

eTable 2. Adjusted Odds Ratios for Neonatal and Early Neonatal Mortality

**List of variables**	**Neonatal**<br>Odds ratio (95% CI)	**Early neonatal**<br>Odds ratio (95% CI)
History of neonatal death		
No*	1	1
Yes	2.23 (1.96 - 2.55)	2.37 (2.05 – 2.72)
Wealth		
Poorest	1.18 (0.86 – 1.62)	1.29 (0.90 – 1.85)
Poorer	1.30 (0.97 – 1.74)	1.39 (1.01 – 1.93)
Middle	1.31 (1.00 – 1.72)	1.35 (0.99 – 1.83)
Richer	1.07 (0.83 – 1.39)	1.09 (0.81 – 1.47)
Richest*	1	1
Mother’s schooling in years		
No Schooling	1.20 (0.94 – 1.53)	1.21 (0.92 – 1.57)
Less than 5 Years	1.46 (1.10 – 1.93)	1.48 (1.09 – 2.01)
5 to 7 years	1.44 (1.14 – 1.82)	1.46 (1.13 – 1.89)
8 to 9 years	1.08 (0.84 – 1.37)	1.03 (0.79 – 1.35)
10 to 11 years	1.39 (1.07 – 1.80)	1.43 (1.07 – 1.91)
12 or more years*	1	1
Frequency of ANC visits		
No Antenatal visits	1.55 (1.08 – 2.23)	1.56 (1.05 – 2.33)
1 to 4 visits	1.14 (0.84 – 1.54)	1.11 (0.79 – 1.55)
5 to 7 visits	1.19 (0.87 – 1.63)	1.19 (0.85 – 1.67)
8 to 9 visits	1.04 (0.73 – 1.48)	0.98 (0.66 – 1.44)
10 or more visits*	1	1
Don’t Know	0.51 (0.24 – 1.10)	0.60 (0.27 – 1.30)
Birth weight		
Not weighed	7.38 (5.90 – 9.25)	8.23 (6.47 – 10.46)
Low weight: mother's recall	4.00 (3.20 – 5.01)	3.79 (2.96 – 4.86)
Low weight: written card	2.22 (1.74 – 2.84)	2.45 (1.88 – 3.19)
Average or more weight: mother's recall	1.91 (1.58 – 2.30)	1.98 (1.61 – 2.42)
Average or more weight: written card*	1	1
Don’t Know/Missing	5.62 (4.36 – 7.25)	5.64 (4.29 – 7.42)
Duration of Pregnancy		
Less than 9 months	3.93 (3.45 – 4.47)	3.92 (3.41 – 4.51)
9 or more months*	1	1
Birth interval (months)		
Less than 18	1.83 (1.60 – 2.10)	1.84 (1.59 – 2.13)
18 to 59*	1	1
59 or more	1.11 (0.95 – 1.28)	1.14 (0.97 – 1.33)
Mother’s Age at Birth		
Less than 18 Years	0.65 (0.32 – 1.33)	0.49 (0.20 – 1.21)
18 to 34 years*	1	1
35 or more years	1.57 (1.30 – 1.89)	1.64 (1.34 – 2.01)
Birth Size		
Average or Larger*	1	1
Small	1.16 (0.99 – 1.37)	1.15 (0.96 – 1.38)
Very Small	2.08 (1.72 – 2.51)	1.96 (1.59 – 2.42)
Don’t Know	1.76 (1.41 – 2.20)	1.94 (1.54 – 2.45)
Religion		
Hindu*	1	1
Muslim	0.88 (0.75 – 1.03)	0.94 (0.80 – 1.12)
Christian	0.91 (0.59 – 1.41)	0.85 (0.52 – 1.38)
Sikh	0.74 (0.40 – 1.35)	0.63 (0.31 – 1.29)
Buddhist / Neo– Buddhist	1.16 (0.56 – 2.38)	1.18 (0.53 – 2.61)

© 2020 Kappor M et al. *JAMA Network Open.*

Other	0.99 (0.57 – 1.72)	0.97 (0.54 – 1.76)
Caste		
Scheduled Caste*	1	1
Scheduled Tribe	0.77 (0.63 – 0.94)	0.78 (0.63 – 0.97)
Other Backward Caste (OBC)	0.97 (0.85 – 1.11)	0.95 (0.83 – 1.10)
Other	0.94 (0.79 – 1.12)	0.95 (0.78 – 1.14)
Don’t Know	0.65 (0.32 – 1.33)	0.52 (0.23 – 1.21)
Residence		
Urban*	1	1
Rural	1.05 (0.89 – 1.23)	1.06 (0.89 – 1.27)
Covered by Health Insurance		
No*	1	1
Yes	1.11 (0.94 – 1.32)	1.12 (0.93 – 1.34)
Toilet Facility		
Flush*	1	1
Pit Latrine	1.08 (0.87 – 1.35)	1.02 (0.80 – 1.30)
Open Defecation	1.11 (0.96 – 1.30)	1.05 (0.89 – 1.24)
Other3	1.26 (0.82 – 1.94)	0.74 (0.44 – 1.25)
Source of Drinking Water		
Piped into dwelling*	1	1
Piped to yard/plot	1.18 (0.91 – 1.54)	1.17 (0.87 – 1.57)
Public tap/standpipe	1.06 (0.83 – 1.37)	1.04 (0.79 – 1.37)
Tube well or borehole	1.17 (0.94 – 1.46)	1.21 (0.95 – 1.54)
Protected well	1.13 (0.77 – 1.65)	1.04 (0.69 – 1.58)
Unprotected well	0.95 (0.69 – 1.30)	0.89 (0.63 – 1.26)
Protected Spring	1.05 (0.63 – 1.75)	0.69 (0.37 – 1.31)
Unprotected Spring	1.23 (0.75 – 2.01)	1.38 (0.81 – 2.35)
River/Dam/Lake/Ponds/Stream/Canal	1.65 (1.12 – 2.43)	1.84 (1.23 – 2.77)
Rainwater	0.91 (0.29 – 2.87)	1.10 (0.35 – 3.47)
Tanker Truck	1.14 (0.64 – 2.02)	1.09 (0.64 – 1.87)
Cart with Small Tank	0.91 (0.18 – 4.66)	0.28 (0.04 – 2.16)
Bottled Water	0.98 (0.49 – 1.98)	0.91 (0.39 – 2.09)
Community RO Plant	1.00 (0.44 – 2.24)	0.96 (0.38 – 2.41)
Other	1.06 (0.32 – 3.53)	1.13 (0.31 – 4.15)
Not a de jure resident	0.95 (0.57 – 1.59)	1.67 (0.91 – 3.08)
Smoke/Tobacco		
No*	1	1
Yes	1.41 (1.21 – 1.65)	1.40 (1.18 – 1.65)
Alcohol		
No*	1	1
Yes	0.94 (0.68 – 1.31)	0.92 (0.64 – 1.31)
Skilled Birth Assistance		
No	0.72 (0.58 – 0.90)	0.79 (0.63 – 1.00)
Yes*	1	1
Delivery at Home or Other		
No*	1	1
Yes	0.59 (0.47 – 0.75)	0.48 (0.38 – 0.62)
Full tetanus protection		
No	1.24 (1.07 – 1.44)	1.27 (1.08 – 1.49)
Yes*	1	1
Took Iron Tablets and Syrup for 100 days or more		
No iron tablets/syrup	1.03 (0.91 – 1.17)	1.02 (0.89 – 1.16)
Less than 100*	1	1
100 or more	1.00 (0.87 – 1.16)	1.01 (0.86 – 1.18)
Don’t know	0.82 (0.48 – 1.38)	0.92 (0.53 – 1.59)
Took drugs for intestinal parasites during pregnancy		
No*	1	1
Yes	1.04 (0.89 – 1.23)	1.00 (0.84 – 1.19)

© 2020 Kappor M et al. *JAMA Network Open.*

Don't know	0.84 (0.48 – 1.50)	0.73 (0.37 – 1.43)
During Pregnancy: Mosquito Net Usage		
Never*	1	1
Sometimes	0.90 (0.78 – 1.02)	0.91 (0.79 – 1.06)
Regularly	0.83 (0.72 – 0.95)	0.88 (0.76 – 1.02)
During Pregnancy: Convulsions not from Fever		
No*	1	1
Yes	1.01 (0.88 – 1.15)	1.01 (0.87 – 1.17)
Don't know	1.11 (0.39 – 3.13)	1.28 (0.44 – 3.70)
During Pregnancy: Swelling of legs, body or face		
No*	1	1
Yes	1.19 (1.06 – 1.33)	1.19 (1.05 – 1.35)
Don't know	0.68 (0.10 – 4.81)	0.72 (0.10 – 5.22)
During Pregnancy: Received Supplementary Nutrition from Anganwadi Centre		
No*	1	1
Yes	0.77 (0.69 – 0.86)	0.83 (0.73 – 0.93)
Getting Medical Help for self: Getting Permission to go		
No Problem*	1	1
Big Problem	1.02 (0.87 – 1.20)	1.08 (.9 – 1.28)
Not a big Problem	1.04 (0.90 – 1.21)	1.09 (0.93 – 1.27)
Getting Medical Help for self: Getting money needed for treatment		
No Problem*	1	1
Big Problem	1.03 (0.88 – 1.22)	0.99 (0.83 – 1.19)
Not a big Problem	0.95 (0.82 – 1.10)	0.93 (0.80 – 1.09)
Getting Medical Help for self: Distance to health facility		
No Problem*	1	1
Big Problem	1.01 (0.82 – 1.24)	1.08 (0.85 – 1.36)
Not a big Problem	1.09 (0.91 – 1.30)	1.12 (0.92 – 1.36)
Getting Medical Help for self: Having to take transport		
No Problem*	1	1
Big Problem	0.99 (0.80 – 1.22)	0.94 (0.75 – 1.19)
Not a big Problem	0.88 (0.73 – 1.05)	0.87 (0.71 – 1.05)
Getting Medical Help for self: Not wanting to go alone		
No Problem*	1	1
Big Problem	0.90 (0.76 – 1.06)	0.92 (0.77 – 1.10)
Not a big Problem	0.98 (0.85 – 1.13)	0.98 (0.84 – 1.14)
Getting Medical Help for self: Concern no female health provider		
No Problem*	1	1
Big Problem	1.04 (0.86 – 1.27)	1.05 (0.85 – 1.29)
Not a big Problem	0.92 (0.77 – 1.10)	0.86 (0.70 – 1.05)
Getting Medical Help for self: Concern no provider		
No Problem*	1	1
Big Problem	0.91 (0.71 – 1.16)	0.83 (0.63 – 1.08)
Not a big Problem	0.99 (0.80 – 1.22)	1.02 (0.81 – 1.29)
Getting Medical Help for self: Concern no drugs available		
No Problem*	1	1
Big Problem	1.02 (0.82 – 1.27)	1.06 (0.83 – 1.35)
Not a big Problem	1.07 (0.87 – 1.32)	1.08 (0.85 – 1.36)
During Pregnancy: Weighed		

© 2020 Kappor M et al. *JAMA Network Open.*

No*	1	1
Yes	1.13 (0.93 – 1.37)	1.16 (0.94 – 1.43)
During Pregnancy: Blood Pressure taken		
No*	1	1
Yes	1.12 (0.90 – 1.38)	1.13 (0.89 – 1.42)
During Pregnancy: Urine Sample taken		
No*	1	1
Yes	0.92 (0.75 – 1.13)	1.03 (0.83 – 1.28)
During Pregnancy: Blood Sample taken		
No*	1	1
Yes	1.27 (1.01 – 1.61)	1.14 (0.89 – 1.46)
Told about Pregnancy Complication: Vaginal bleeding		
No*	1	1
Yes	0.98 (0.81 – 1.19)	0.97 (0.78 – 1.20)
Told about Pregnancy Complication: Convulsions		
No*	1	1
Yes	0.92 (0.76 – 1.12)	1.00 (0.81 – 1.23)
Told about Pregnancy Complication: Prolonged Labour		
No*	1	1
Yes	0.82 (0.67 – 1.01)	0.92 (0.73 – 1.15)
Told about Pregnancy Complication: Severe Abdominal Pain		
No*	1	1
Yes	1.27 (1.03 – 1.57)	1.14 (0.91 – 1.43)
Told about Pregnancy Complication: High Blood Pressure		
No*	1	1
Yes	1.04 (0.87 – 1.23)	1.02 (0.84 – 1.23)
Past pregnancy ended in abortion, miscarriage or stillbirth		
No*	1	1
Yes	0.99 (0.86 – 1.13)	1.01 (0.87 – 1.17)
Birth Order		
2 to 3*	1	1
4 to 6	1.04 (0.92 – 1.18)	1.00 (0.87 – 1.14)
7 or more	1.17 (0.91 – 1.49)	1.07 (0.82 – 1.40)
Mother’s Height		
Less than 145 cm*	1	1
145 to 149.9 cm	0.85 (0.72 – 0.99)	0.90 (0.76 – 1.07)
150 to 154.9 cm	0.91 (0.78 – 1.05)	0.93 (0.79 – 1.10)
155 to 159.9 cm	0.68 (0.56 – 0.82)	0.73 (0.59 – 0.89)
160 or more cm	0.71 (0.55 – 0.93)	0.70 (0.53 – 0.93)
Refused	0.88 (0.18 – 4.39)	0.83 (0.15 – 4.74)
Other	0.78 (0.11 – 5.61)	1
Missing	2.24 (0.53 – 9.52)	1.83 (0.37 – 9.08)
BMI Cut off		
Less than 16.5	1	1
16.5 to 18.49	0.93 (0.74 – 1.16)	0.92 (0.72 – 1.18)
18.5 to 24.99	1.02 (0.83 – 1.25)	1.06 (0.84 – 1.33)
25 or more	1.30 (1.02 – 1.65)	1.30 (1.00 – 1.70)
Flagged	1.06 (0.33 – 3.40)	1.11 (0.30 – 4.11)
Hypertension[iii]		
No*	1	1

[iii] Hypertensive: Average of the 2nd and 3rd reading – Systolic more than 140mm of Hg, and/or Diastolic more than 90mm of Hg.

Yes	1.30 (1.08 – 1.56)	1.35 (1.11 – 1.64)
Missing/Refused/Technical Problem	0.85 (0.63 – 1.14)	0.79 (0.58 – 1.08)
**Anaemia**[iv]		
Severe	2.03 (1.43 – 2.89)	2.08 (1.45 – 2.98)
Moderate	1.34 (1.16 – 1.54)	1.36 (1.16 – 1.59)
Mild	0.97 (0.87 – 1.08)	1.01 (0.89 – 1.13)
None*	1	1
Missing	1.50 (0.12 – 19.44)	1.42 (0.08 – 25.12)
Blood sugar		
140 mg/dl or less	1	1
More than 140 mg/dl	1.64 (1.32 – 2.03)	1.79 (1.43 – 2.24)
Refused/Other/Not tested	0.67 (0.05 – 8.27)	0.90 (0.05 – 15.03)
Missing	0.42 (0.04 – 4.39)	0.47 (0.03 – 6.28)
Delivery by Caesarean Section		
No*	1	1
Yes	1.54 (1.29 – 1.83)	1.45 (1.19 – 1.76)
Sex of Child		
Male*	1	1
Female	0.92 (0.83 – 1.02)	0.89 (0.80 – 0.99)
State fixed effects	Included	Included

[iv] Anaemia adjusted for altitude and for smoking status. Severe: Hemoglobin less than 7 grams per deciliter (g/dL). Moderate: 7 to 9.9 g/dL. Mild: 10 to 10.9 g/dL. None: 11 or more g/dL.

© 2020 Kappor M et al. *JAMA Network Open.*

eTable 3. Maternal History of Neonatal Death and the Attributable and Population–Attributable Risk of Mortality

	Dead (N = 3712)	Alive (N = 187 186)	Relative Risk (95% CI)	Attributable risk percentage (95% CI)	Population attributable risk (PAR)
No history of neonatal death	3080 (82.97%)	176 442 (94.26%)	1.0		
History of neonatal death	632 (17.03%)	10 744 (5.74%)	3.24 (2.98 – 3.52)	69.12% (66.43% – 71.59%)	11.77%

For the purpose of calculating population attributable risk, we used data on all 190,898, most recent livebirths from the NFHS–4 data from India for 2015–16, which includes multiple livebirths, and also livebirths of nulliparous mothers.

© 2020 Kappor M et al. *JAMA Network Open.*
